# A case of acute respiratory distress syndrome due to lymphography with Lipiodol for chylothorax after esophagectomy

**DOI:** 10.1186/s40792-018-0560-y

**Published:** 2019-01-03

**Authors:** Yusuke Taki, Shinsuke Sato, Katsunori Suzuki, Erina Nagai, Masaya Watanabe, Yuichiro Shishido, Nobuaki Nakajima, Masakazu Takagi

**Affiliations:** 1 Department of Gastroenterological Surgery, Shizuoka General Hospital, 4-27-1 Kita Ando Aoi-ku, Shizuoka City, 420-8527 Japan; 2 Department of Respiratory Medicine, Shizuoka General Hospital, Shizuoka City, Japan; 3 Department of Radiology, Shizuoka General Hospital, Shizuoka City, Japan

**Keywords:** Lymphography, ARDS, Lipiodol, Esophagectomy

## Abstract

**Background:**

Lymphography with Lipiodol is useful for chylothorax. There were many slight complications, but reports of acute respiratory distress syndrome (ARDS) after lymphography were few.

**Case presentation:**

A 75-year-old man with esophageal cancer developed chylothorax after esophagectomy. Conservative treatment was ineffective, and he underwent lymphography with 8.5 mL of Lipiodol. He developed a high fever soon after lymphography, followed by severe ARDS requiring artificial respiration 5 days later. He recovered from ARDS but subsequently developed pulmonary fibrosis and was discharged with domiciliary oxygen therapy 3 months later.

**Conclusion:**

Although ARDS is a rare complication of lymphography with Lipiodol, this procedure should be applied carefully in patients with chylothorax.

## Background

Chylothorax is a relatively rare but potentially life-threatening complication after thoracic surgery [1], with a reported incidence of 0.6–3.9% after esophagectomy [1–3]. Lymphography with Lipiodol is useful for both diagnosing and treating chylothorax [4]. To the best of our knowledge, only three cases of acute respiratory distress syndrome (ARDS) due to lymphography with Lipiodol have been reported [5, 6]. We report a patient who developed severe ARDS after Lipiodol lymphography for chylothorax, with a subsequent complication of pulmonary fibrosis.

## Case presentation

A 75-year-old Japanese man presented with a 1-month history of epigastric discomfort. He had a medical history of pulmonary tuberculosis treated with antitubercular agents, but his respiratory function tests were normal. On close examination, he was diagnosed with squamous cell carcinoma with a basaloid carcinoma-like component of the esophagus. The preoperative diagnosis was clinical T3N0M0 stage IIA lower esophageal cancer, according to the Union for International Cancer Control, seventh edition. The patient underwent preoperative chemotherapy (5-fluorouracil and cisplatin), but a second preoperative course was canceled because of the deterioration of his renal function to creatinine 1.34 mg/dL after the first course. He underwent video-assisted thoracoscopic esophagectomy in the left lateral position with three-field lymph node dissection and hand-assisted laparoscopic surgery. His thoracic duct was preserved without apparent injury. The surgical time was 394 min, and the intraoperative blood loss was 430 ml. Tube feeding was started from the second postoperative day. Thoracic drain fluid increased to 600 ml on postoperative day 5, and its appearance became milky. Pleural effusion triglyceride levels were 111 mg/dl. The patient was diagnosed with chylothorax, and enteral nutrition was discontinued. However, despite total parenteral nutrition (TPN), the chylothorax continued, and pleural effusion increased to > 1500 ml/day. He underwent lymphography for diagnostic and therapeutic purposes on the ninth postoperative day.

**Fig. 1:**
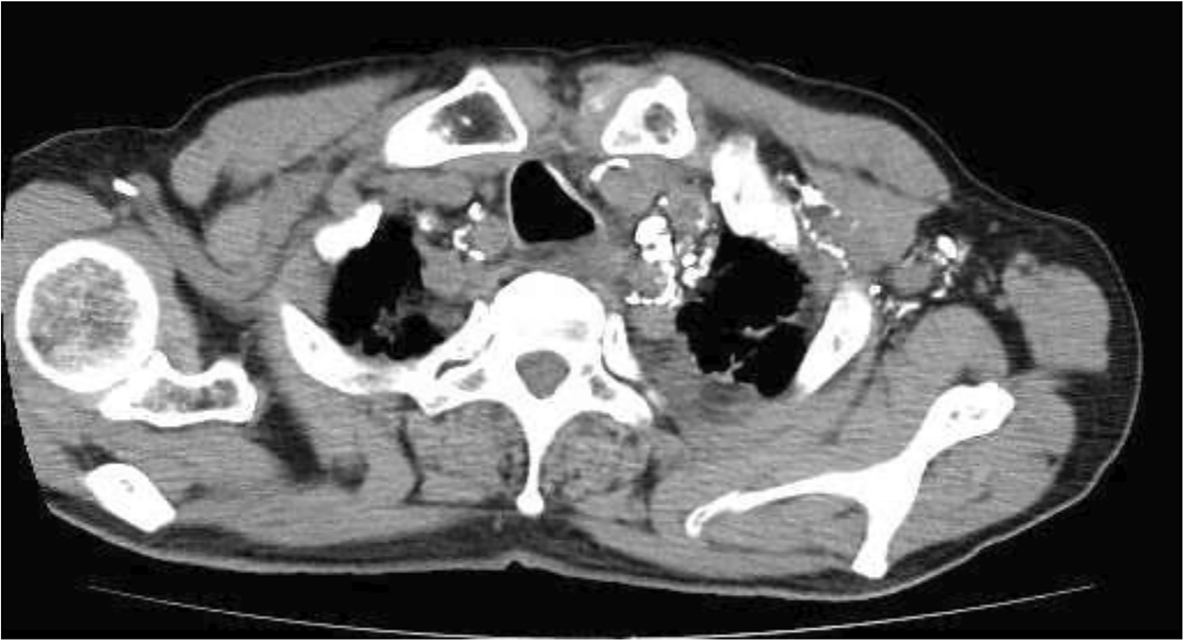
Computed tomography image showing enhanced supraclavicular lymphatic vessels

We injected patent blue subcutaneously into the left acrotarsium, under local anesthesia. The lymphatic vessels were visualized, and we inserted a 27-gauge needle into one of the lymphatic vessels and injected Lipiodol at 0.1 ml/min. Videofluoroscopy showed lymphatic vessel enhancement up to the pelvis after 5 ml of Lipiodol, and a further 3.5 ml was injected at 0.14 ml/min. Computed tomography (CT) showed enhanced supraclavicular lymphatic vessels (Fig. 1), but no lymphatic leakage was detected. No sign of aspiration was observed during lymphography.

**Fig. 2:**
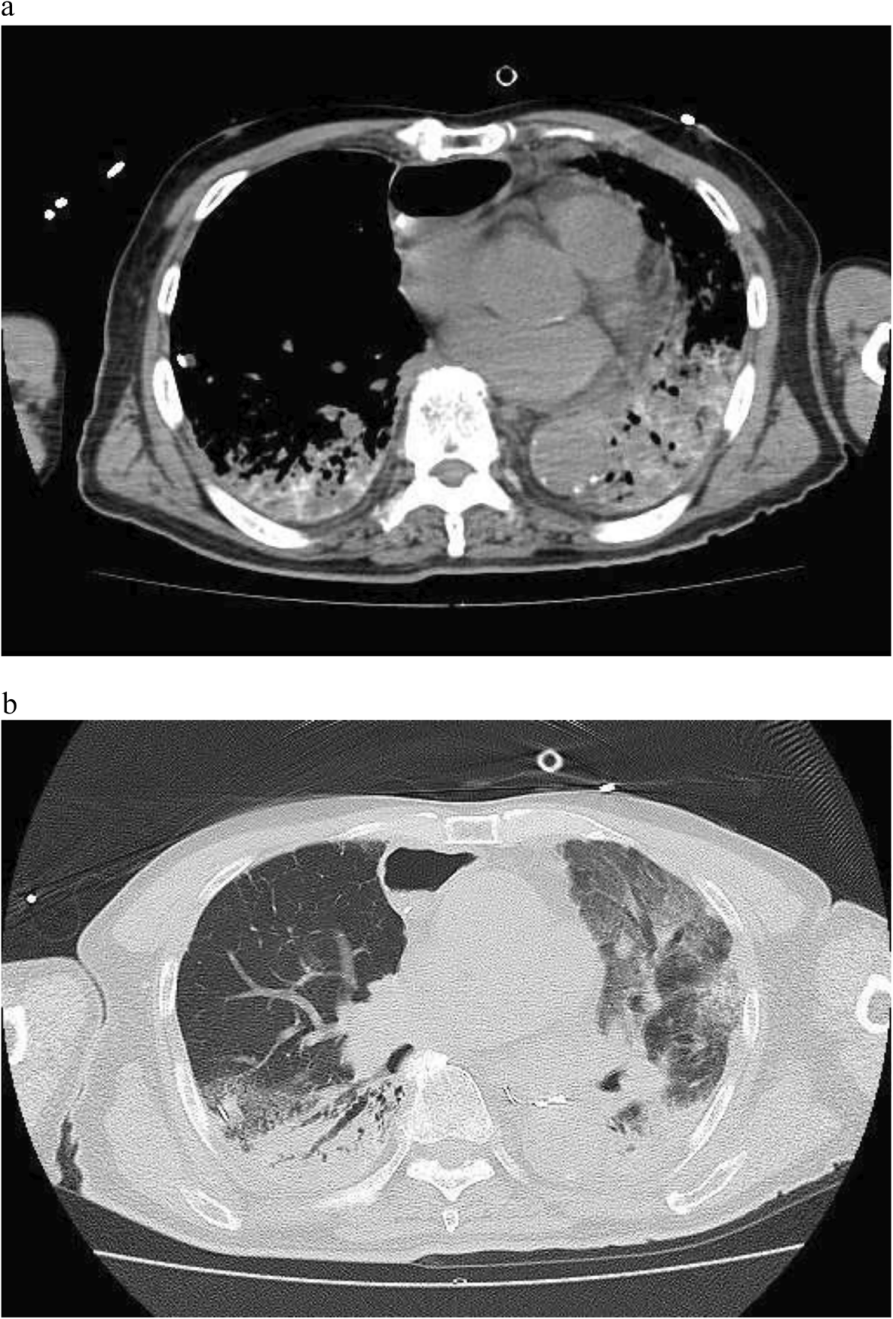
Computed tomography image showing high-density substance in the lung (**a**) and ground-glass opacity in bilateral lungs (**b**)

**Fig. 3:**
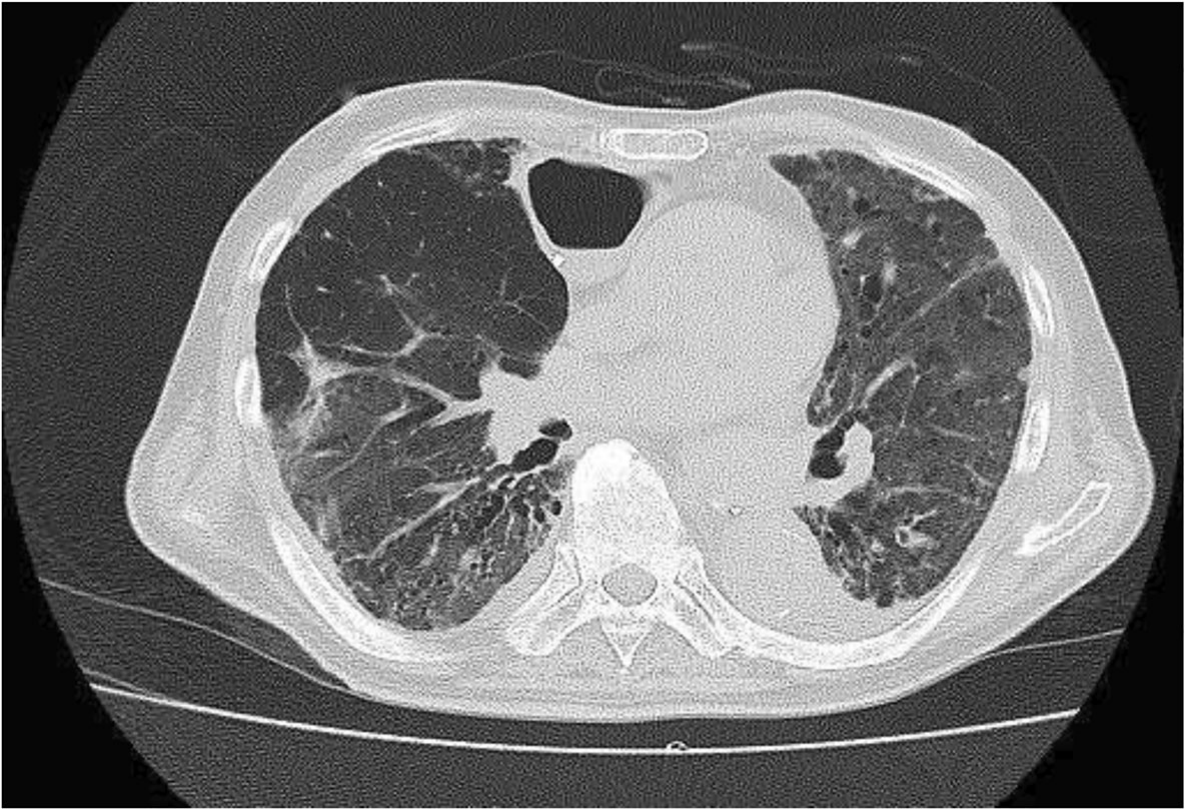
Computed tomography image showing bilateral pulmonary fibrosis

The patient developed chills, a fever (39 °C), and hypoxia 2 h after lymphography and was treated with oxygen administration and piperacillin-tazobactam for Lipiodol pulmonary embolism and prevention of secondary pneumonia. His hypoxia improved but intermittent fever continued after 4 days. Though his pleural effusion decreased to 200 ml/day on the 11th postoperative day, his hypoxia deteriorated suddenly on the 14th postoperative day, requiring artificial respiration. Blood gas analysis showed PaO_2_ 85.3 mmHg with FiO_2_ 0.6 and positive end-expiratory pressure 12 cmH_2_O. CT revealed high-density substance in the lung (Fig. 2a) and bilateral ground-glass opacity (Fig. 2b). We diagnosed severe ARDS, according to the Berlin definition, and started sivelestat sodium hydrate and lung-protective ventilation with low tidal volumes and prone position. Prednisolone 20 mg was added on the 21st postoperative day to prevent pulmonary fibrosis, without success, and he underwent a tracheotomy on the 28th postoperative day. He was weaned from mechanical ventilation on the 50th day but still required oxygen. He was finally discharged on the 112th day, with domiciliary oxygen therapy for hypoxemia due to pulmonary fibrosis (Fig. 3).

## Discussion

This case highlights two important clinical issue: first, lymphography with even in the amount of less than 10 ml can cause ARDS, and second, Lipiodol-induced ARDS can cause severe pulmonary fibrosis.

Lipiodol (iodine organically combined with ethyl esters of poppy seed fatty acids) is a long-standing medical contrast agent. Japanese package inserts suggest that the appropriate dosage for unilateral lymphography from the lower extremities is 10 ml. Dolan et al. reported complications in 166 of 522 cases (31.8%) of lymphography [7], including fever (18.6%), nausea/vomiting (4.4%), pain (3.3%), and respiratory signs/symptoms (1.3%). Although most complications were slight and transient, two patients experienced severe cardiovascular difficulties. Seven patients developed respiratory signs and symptoms, including chest tightness, dyspnea, cough, and wheezing, but ARDS was not noted. We searched PubMed from 1946 to 2018 using the keywords “lymphangiography” and “respiratory” and found only three reports of ARDS after lymphangiography in the English published literature [5, 6]. Goff and Gaensler reported a case of respiratory distress syndrome following lymphangiography and retroperitoneal lymph node dissection [5] in a patient who received 20 ml of ethiodized oil injected into each leg. The respiratory distress syndrome was so severe that the patient required artificial ventilation and hydrocortisone. Their hypoxemia improved after 26 days, but mild restrictive ventilatory impairment and reduced diffusing capacity were still evident at discharge. Silvestri et al. reported two cases of respiratory distress syndrome due to lymphangiography [6], both of whom underwent bipedal lymphography with approximately 10 ml of ethiodized oil. These previous and current cases all demonstrated high fever, suggesting that the ethiodized oil acted as an inflammatory substance.

Gold et al. reported that the mechanism of pulmonary injury due to lymphography was a reduction of diffusing capacity and pulmonary capillary blood volume [8]. These signs initiated between 3 and 13 h and lasted until a maximum of 256 h. Silvestri et al. described the pathophysiology of oil inflammation in rats [6]. They showed that small numbers of polymorphonuclear leukocytes and mononuclear phagocytes were present around the lipid and within the interstitium at 4 h after injection of ethiodized oil, and a mild multifocal interstitial inflammatory response developed 1 day after injection. This inflammatory response became more extensive, with flooding of the air spaces with hemorrhage and edema. These responses had completely disappeared by 6 weeks. In our case, acute exacerbation of symptoms was observed 5 days after onset. This clinical course indicated pulmonary embolism of Lipiodol as a cause. We considered that Lipiodol deposited in the lung was chronically inflamed, immune system reacted, and the respiratory condition worsened rapidly 5 days later which led to ARDS.

The current patient developed severe pulmonary fibrosis after ARDS, despite steroid administration. The indication of steroids for ARDS is controversial, and patients with ARDS for > 14 days should not receive steroids because methylprednisolone was shown to increase 60-day mortality [9]. In patients with early ARDS (< 72 h), however, glucocorticoids reduced the duration of mechanical ventilation, and the length of intensive care unit stay and mortality [10]. The current patient received prednisolone on the seventh day after the onset of ARDS. Although fibroblast migration and collagen deposition have been recognized as late events, fibroproliferation and collagen deposition start at the onset of ARDS [11], suggesting that earlier administration of steroids might prevent lung fibrosis.

Chylothorax is a life-threatening complication after esophagectomy, and conservative treatments such as thoracostomy drainage, low-fat diet, nil per os (NPO), and TPN should be administered before resorting to lymphography. Marts et al. reported that chylothorax was resolved by conservative treatment in 79% of patients [12]. Octreotide [13] and etilefrine [14] were also reported to be effective for chylothorax, and a pleurodesis is a conservative option if NPO is ineffective [15]. Thoracic duct ligation is also an effective therapy, and the success rate is now 85–100% [3, 16]. Even if the location of the lymphatic leakage is not detected during surgery, ligation of the lymphatic duct just above the diaphragm improves chylothorax [1]. Reisenauer et al. reported an algorithm for the management of postoperative chylothorax [16], including surgical thoracic duct ligation if the chest tube output is > 1100 ml after NPO and TPN. If surgical ligation is ineffective, they advocated lymphangiography and thoracic duct embolization. We chose lymphography before surgical ligation because we considered that lymphography was less invasive than surgery and the location of lymphatic leakage could be identified. Given that our case presented with a rare but critical complication of Lipiodol lymphography, surgical ligation was an appropriate prevenient treatment strategy when the chest tube output exceeded 1100 ml after NPO.

## Conclusions

We encountered a patient who developed severe ARDS due to lymphangiography of Lipiodol for chylothorax after esophagectomy. ARDS due to lymphangiography is a rare but critical complication, and there is a range of treatment options for chylothorax. The indications of lymphography for chylothorax should be considered carefully.

## Abbreviations

ARDS: Acute respiratory distress syndrome
CT: Computed tomography
NPO: Nil per os
TPN: Total parenteral nutrition
